# Hemogregarines in bufonid anurans from the Brazilian Amazon, with description a new species of *Lankesterella* (Apicomplexa: Lankesterellidae)

**DOI:** 10.1016/j.ijppaw.2025.101044

**Published:** 2025-02-04

**Authors:** Tássio Alves-Coêlho, Darlison Chagas-de-Souza, Carolina Romeiro Fernandes Chagas, Germán Alfredo Gutiérrez-Liberato, Lívia Perles, Amir Alabi, Marcos Rogério André, Lúcio André Viana

**Affiliations:** a Laboratory of Morphophysiological and Parasitic Studies, Department of Biological and Health Sciences, Federal University of Amapá, 68903-419, Macapá, Amapá, Brazil; b Postgraduate Program in Tropical Biodiversity, Federal University of Amapá, 68903-419, Macapá, Amapá, Brazil; c P. B. Šivickis Laboratory of Parasitology, Nature Research Centre, Akademijos 2, 08412, Vilnius, Lithuania; d Vector-Borne Bioagents Laboratory (VBBL), Department of Pathology, Reproduction and One Health, School of Agricultural and Veterinarian Sciences, São Paulo State University (UNESP), 14884-900, Jaboticabal, São Paulo, Brazil

**Keywords:** Hemoparasites, Amphibians, Hemogregarines, Haemococcids, Integrative taxonomy

## Abstract

Here we describe, in an unprecedented way for the Brazilian Amazon, one Apicomplexa species infecting an anuran in Pará, Brazil, using an integrative approach that includes taxonomy, providing morphology, morphometrics, and molecular data. Samples were collected between February 2021 and February 2022 in the community of Curupira, municipality of Santarém, and in the industrial area of Marabá, both in Pará state. Specimens of *Rhinella marina* and *Rhinella diptycha* were captured by hand during the active search, and blood samples were collected by cardiac puncture. One blood aliquot was used to prepare blood smears for microscopical analysis and another aliquot for further molecular analyses. Of the five *R. marina* caught, one (20%) presented parasites morphologically compatible with the parasites belonging to the genus *Hepatozoon*, while of the three *R. diptycha* caught, one (33.3%) presented parasites morphologically compatible with the parasites belong to the genera *Lankesterella* and *Hemolivia*. Based on molecular data of *18S* rDNA sequences, the *Hepatozoon* sequence obtained clustered with other parasite species recovered from Brazilian amphibians. The *Lankesterella* sp. sequence was placed in a sister clade of *Lankesterella* species described in birds and close to *L. minima*, described in amphibians. All attempts to amplify *Hemolivia* parasite DNA were unsuccessful. We described *Lankesterella oliviacatarinae* n. sp. and reported infection by *Hepatozoon* sp. and *Hemolivia* sp. in bufonids anurans from the Brazilian Amazon. This study increases knowledge of Brazilian anuran hemoparasites and confirms the importance of using an integrative approach for the taxonomy of these parasite groups.

## Highlights

• First description of a *Lankesterella* species found in Amazonian amphibians using integrative taxonomy.
• Hemoparasites in common anuran species have yet to be described.
• Advances in the knowledge of the classification of hemoparasites in Brazilian amphibians.
• New distribution and host record for *Hemolivia*.

## 1 Introduction

Brazil has the highest diversity of amphibian species in the world, with 1188 species, of which 1144 are anurans (Segalla et al., 2021). Amphibians are an important component of global biodiversity, found in most biomes and act in the food chain as carnivores, herbivores, predators and prey (Ramalho et al., 2019). However, with several species at risk of extinction, amphibians are considered one of the most threatened vertebrate groups, which are subjected to several threats, such as: habitat fragmentation and elimination, roadkill, and diseases caused by different infectious agents (Marcogliese et al., 2009; Rollins-Smith, 2017; González-del-Pliego et al., 2019).

Although one of the most studied pathogens is the fungus *Batrachochytrium dendrobatidis* (Bd), a pathogen responsible for chytridiomycosis in amphibians and the cause of population losses in several parts of the world (Amorim et al., 2019; Prado et al., 2023), amphibians can also harbour a wide variety of endoparasites and ectoparasites (Kohn and Fernandes, 2014; Campião et al., 2014; Coêlho et al., 2019, 2021a, 2021b; Oliveira et al., 2020; Úngari et al., 2020, 2021, 2022; Netherlands et al., 2020a, 2020b; Mendoza-Roldan et al., 2020). It is known that these parasites can affect their behavior, fitness, feeding, reproduction and fertility (Bower et al., 2019). Focusing on anurans’ hemoparasites, intracellular apicomplexans of the genus *Hepatozoon* Miller 1908 (Apicomplexa: Adeleorina) have been frequently recorded (Menezes Leal et al., 2015; Úngari et al., 2021) while *Lankesterella* Labbé 1899 (Apicomplexa: Eimeriorina) (Costa and Pereira, 1971; Lainson and Paperna, 1995) and *Hemolivia* Petit, Landau, Baccam and Lainson (1990) (Apicomplexa: Adeleorina) (Petit et al., 1990) seem to be less common.

*Hepatozoon* parasites have a complex life cycle involving a blood-sucking invertebrate vector (e.g. ticks, mosquitoes, fleas, and lice), which acts as definitive hosts. After the blood meal in an infected vertebrate host, gamonts leave the infected erythrocytes and the sexual cycle of the parasite takes place in the intestine or Malpighian tubules of the vector, resulting in the subsequent formation of oocysts, which contain hundreds of sporocysts with infective sporozoites (Smith, 1996; Baneth et al., 2007). The transmission to the vertebrate intermediary hosts occurs when it ingests an infected vector or paratenic vertebrate (i.e. amphibians, fishes) hosts with cystozoites in tissues (Smith, 1996; Baneth et al., 2007).

Although *Hepatozoon* parasites are frequently reported in anurans from other continents (Barta and Desser, 1984; Levine, 1988; Netherlands et al., 2014, 2018; Conradie et al., 2017), only four species have been described in Brazil: *Hepatozoon leptodactyli* (Lesage 1908; Pessoa 1970, infecting *Leptodactylus* species, described only based on morphological and morphometric data; and recently, Úngari et al. (2021) described *Hepatozoon formosus* Úngari, Netherlands, Silva and O'Dwyer (2021) and *Hepatozoon longinucleus* Úngari, Netherlands, Silva and O'Dwyer (2021) both infecting *Leptodactylus labyrinthicus*; and *Hepatozoon latrensis* Úngari, Netherlands, Silva and O'Dwyer (2021) found infecting *Leptodactylus latrans*, all in the state of Mato Grosso do Sul, in the Cerrado biome. However, despite the higher diversity of amphibians in the Amazon region (>300 species) (Segalla et al., 2021), there are no descriptions data for *Hepatozoon* species that infecting them.

*Lankesterella* is another apicomplexan parasite found in anurans, but with fewer records in Brazil (Costa and Pereira, 1971; Lainson and Paperna, 1995). Parasites belonging to the genus *Lankesterella* share certain characteristics with the hemogregarines, as the presence of mobile stages in circulating blood cells (Desser, 1993). They are monoxenous parasites, with the sporogony, merogony and gametogony stages occurring in the tissues of vertebrate hosts during their life cycle, and the parasites do not replicate in their invertebrate hosts, which act as paratenic vector (Desser, 1993; Chagas et al., 2021b), which can acquire the parasite during a blood meal. The transmission occurs by sporozoite inoculation during intermediate host feeding (leeches) or ingestion of infected vectors (mites or mosquitoes) (Desser, 1993; Chagas et al., 2021b). However, the life cycle of these parasites has not been completely elucidated, and only inferences based on *Lankesterella* parasites in birds have been made given the results obtained during experimental infections of vectors, PCR-based investigations, and histological findings (Desser, 1993; Chagas et al., 2021b; Keckeisen et al., 2024).

Relatively, little is known about lankesterellids in amphibians. One of the first intraerythrocytic parasites mentioned is *Lankesterella minima* by Chaussat (1850) in France, infecting *Pelophylax* kl. *esculentus* (Linnaeus, 1758), who first named the parasite *Anguilina minima* (Barta et al., 2001). For several years, various species were described in the blood of birds as *Hepatozoon* and *Atoxoplasma*, based solely on morphology and morphometry. In 1899, Labbé described the genus *Lankesterella*, and the previously described *Lankesterella* and others Apicomplexa species were regrouped in the genus *Hepatozoon*, demonstrating that this is still a poorly studied group with limited information available, particularly for amphibians (Costa and Pereira, 1971; Lainson and Paperna, 1995; Merino et al., 2006; Megía-Palma et al., 2017; Martínez et al., 2018; Tomé et al., 2021; Chagas et al., 2021a, 2021b).

Currently, ten species of *Lankesterella* are known to infect amphibians, most of which have only been described by morphology, with two species reported in Brazil: *Lankesterella* *petiti* Lainson and Paperna (1995) in *Rhinella marina* (Linnaeus 1758) of Belém, Pará state and *Lankesterella alencari* Costa and Pereira (1971) in *Leptodactylus latrans* Chaussat (1850) of Rio de Janeiro, state of Rio de Janeiro (Costa and Pereira, 1971; Lainson and Paperna, 1995). Only two decades ago, Barta et al. (2001) obtained the first genetic sequence for a lankesterellid, characterizing *18S* rDNA gene of *L. minima*. However, in the São Paulo state, Brazil, only one study used molecular-based methods for the detection of *Lankesterella* sp. in *Rhinella diptycha* (Cope, 1862), but without morphological analysis (Ferreira et al., 2020).

Parasites belonging to the genus *Hemolivia* are registered infecting species of amphibians and reptiles. The merogony occurs in the blood of the vertebrate hosts (intermediate hosts). The fertilization, formation of oocysts and sporocysts with sporozoites occurs in the tissues of the definitive hosts (ticks). Ticks are known vectors for *Hemolivia*, where after feeding on an infected host, they need to be ingested for the infection that occur in the vertebrate hosts (Petit et al., 1990). To date, there have been only two records of *Hemolivia* in amphibians based on morphological and morphometric data, namely *Hemolivia stellata* Petit et al. (1990) in *R. marina* and *Hemolivia* sp. infecting *Rhinella major* (Müller and Hellmich, 1936) from Belém and Santarém, respectively, both in Pará state, Brazil (Petit et al., 1990; Oliveira et al., 2020).

Given the fragmented information available in the literature, the application of an integrative approach in the studies targeting Apicomplexa parasites is essential. This is even more important for the ones targeting anurans from the Brazilian Amazon, given the lack of studies that have been conducted as far. The present study aimed to (i) morphologically describe and molecularly characterize a novel *Lankesterella* species infecting anurans collected in the Pará state, Brazil; (ii) to recorded *Hepatozoon* and *Hemolivia* infections in bufonids anurans; and (iii) to highlight the importance of combining classical and modern parasitology to investigate and describe new species of Apicomplexa parasites.

## 2 Material and methods

### 2.1 Sampling area and anuran capture

**Fig. 1:**
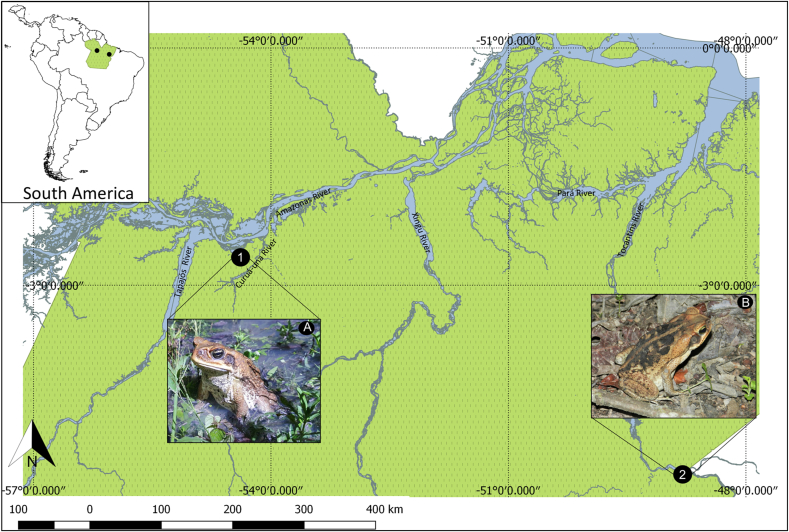
Map of anuran collection sites and specimens captured in Pará state, Brazil. (**A)** Specimen of *Rhinella marina*; **(B)** Specimen of *Rhinella diptycha*; **(1)** Curupira community, in Santarém Municipality; **(2)** Industrial area of the Municipality of Marabá.

Between the months of February 2021 and February 2022, five specimens of *R. marina* (Fig. 1A) were collected through active search on the Curupira community, in Santarém (2°40′46.37"S, 54°32′20.95″ W) (Fig. 1) and three specimens of *R*. *diptycha* (Fig. 1B) on the industrial area of Marabá (5°25′2.83″ S, 49°6′27.87″ W) (Fig. 1), both in Pará state, Brazil.

The specimens were individually separated in plastic bags and transported to the field laboratory where body mass (W; in grams) and snout-vent length (SVL; in millimeters) were recorded and presented with mean, standard deviation and minimum and maximum values. After that, blood samples were collected by cardiac puncture using hypodermic 1 mL syringes. A few drops of blood were used to prepare two thin blood smears that were stained using the Fast-Panoptic method (Laborclin® Brazil). The remaining blood was stored in tubes containing 96% ethanol for further molecular analysis. After the sampling, the individuals were released on the same collection site.

### 2.2 Ethical approval

All the procedures were approved and performed following the principles adopted by the Brazilian College of Animal Experimentation - COBEA. This study was approved by the Animal Use Committee of the Universidade Federal do Amapá (authorization # 011/2021). The licenses for the collection of amphibians used in the present study were granted by the Brazilian Institute for the Environment and Renewable Natural Resources through authorizations issued by the Biodiversity Information and Authorization System - SISBIO nº 77466-1. For access to genetic heritage, this study was authorized by the National System for the Management of Genetic Heritage and Associated Traditional Knowledge (protocol # AC5C1B1).

### 2.3 Blood smear analysis: morphological and morphometric characterization of hemoparasites

The microscopic analysis of the thin blood smears was conducted using a Zeiss Axiostar Plus and consisted of screening 100 fields at 400 × and 1000× magnification. All hemoparasites found were photographed with a Zeiss Axioplan optical microscope with an Axiocam ERc 5S camera. The Zen Blue 2 software package was used to obtain morphometric data.

*Lankesterella* morphometry was adjusted according to the parasite morphotype or stage and consisted of the length and width of the parasitophorous vacuole (PV), if present; the length and width of the parasite when visible (if not covered by a PV); and the length and width of the parasite nucleus, if measurable. Width measurements and PV length were taken across the longest and widest points. Measurements of the length of the parasites were taken across the center of the parasite, following the curve, if the parasites were curved. *Hepatozoon* and *Hemolivia* were measured according to Oliveira et al. (2020). The similarity between measurements of intracellular gamonts of the described *Hepatozoon* species were calculated using the Bray-Curtis index. All measurements are provided in micrometers (μm) with mean, standard deviation, and range. The effect of the parasite on the erythrocytes was evaluated by the comparison of length and wide of infected and non-infected cells using the nonparametric Mann-Whitney Test, with a significance level of 5%.

The parameters of prevalence and intensity of infection were calculated according to Bush et al. (1997) using the software Quantitative Parasitology 3.0 (Reiczigel et al., 2019). To estimate the mean infection intensity (expressed as the number of parasites/mL) the direct method was used, with some adaptations from Carli (2001). Briefly, all the parasites found in 100 microscopic fields with a magnification of 1000 × were recorded and calculated assuming that 100 microscopic fields are equivalent to 0.2 μl of blood, intensity of infection = (number of parasites × 5) × 1.000 = (parasites/mL).

### 2.4 DNA extraction, PCR, and sequencing analysis

Only blood samples that were microscopically positive were subjected to DNA extraction, which was performed from 50 μL of ethanol preserved blood using Biopur Kit Mini Spin Plus® (Mobius, Brazil), according to the manufacturer's instructions.

After being extracted, DNA was used for conventional PCR amplification. The PCR reactions have been performed for the detection of *Hepatozoon* and *Lankesterella*, targeted a fragment of ∼1200 bp of the nuclear *18S* rDNA gene using the primers HepF300 (5′-GTT TCT GAC CTA TCA GCT TTC GAC G-3′) and ER (5′-CTT GCG CCT ACT AGG CAT TC-3′) (Ujvari et al., 2004; Kvičerová et al., 2008). PCR conditions were as follows: initial denaturation at 95 °C for 3 min, followed by 35 cycles of 95 °C for 30 s, with an annealing temperature of 60 °C for 30 s, and an extension of 72 °C for 2 min; and following the cycles a final extension step of 72 °C for 10 min. In addition, the primers HepF300 and HepR900 (Ujvari et al., 2004) and HEMO1 and HEMO2 (Perkins and Keller, 2001) were used to detect *Hemolivia* parasite, following the protocols described in the original papers (Perkins and Keller, 2001; Ujvari et al., 2004).

PCR reactions were performed in a total volume of 25 μL, using 12.5 μL OneTaq® 2X Master Mix with Standard, 1.25 μL (10 μM) of each primer, and at least 25 ng of DNA. The final reaction volume was reached by adding PCR-grade nuclease-free water (Thermo Scientific). Ultra-pure sterile water (Life Technologies®, Carlsbad, CA, USA) was used a negative control in all PCR assays and for the positive control, DNA from *Hepatozoon* sp. (GenBank accession number MK503648) detected in naturally infected amphibians was used. The success of amplification was analysed in 1% agarose gel stained by ethidium bromide solution and visualized in a UV transilluminator (ChemiDoc MP Imaging System, Bio Rad®). Positive amplifications were purified using ExoSAP-IT™ PCR Cleanup (ThermoFisher®) and sent for sequencing in both directions. The sequencing was performed by Sanger method using ABI PRISM 3730 DNA Analyzer (Applied Biosystems) at Human Genome and Stem Cell Research Center, “Instituto de Biociências”, University of São Paulo (USP), Brazil.

Obtained sequences were evaluated and edited using BioEdit V. 7.2.5 (Hall, 1999). Both strands were aligned to obtain a consensus sequence, which was compared to the other sequences deposited in GenBank using the BLASTn (Benson et al., 2018). The sequences obtained were deposited in GenBank (accession numbers PP340976 and PQ283679).

### 2.5 Phylogenetic analyses

The phylogenetic inferences for *Hepatozoon* and *Lankesterella* were constructed separately using both Bayesian Inference (BI) and Maximum likelihood (ML). The BI was carried out with MrBayes V. 3.2.7 (Ronquist and Huelsenbeck, 2003). Obtained sequences were aligned with other sequences deposited in GenBank using MAFFT software online version 7 (Katoh et al., 2019). The alignment for *Hepatozoon* consists of 57 sequences and 1199 bp. The *18S* rDNA sequences from *Adelina*, *Klossia*, and *Klossiella* were used as an outgroup. The alignment for *Lankesterella* consists of 72 sequences and 1354 bp; DNA sequences from 12 different Apicomplexa genera were used, and Haemosporida sequences were used as an outgroup, as described in (Chagas et al., 2021b). In both cases, the best-fit substitution model GTR was selected by MrModelTest2 V. 2.3 (Nylander, 2023). For both parasites, each run was conducted with four chains and with a sampling frequency of every 100th generation over 3 million generations. We discarded 25% of the trees as ‘burn-in’. The remaining trees were used to construct a consensus tree. The ML analyses were conducted using PhyML implemented in Geneious Prime v2025.0.2 (www.geneious.com) using the same alignment and model mentioned above. Nodal support values were calculated using 1000 bootstrap replicates. The trees generated by BI and ML were used to construct a consensus tree, separated for each parasite using Geneious Prime v2025.0.2. Posterior probability and bootstrap values were considered as in the original trees. The phylogenetic trees were visualized using FigTree V. 1.4.0 (Rambaut, 2023). The sequence divergence was calculated for *Hepatozoon* and *Lankesterella* separately using Jukes-Cantor model of substitution, with all substitutions weighted equally (uniform rates), implemented in MEGA V. X (Kumar et al., 2018).

## 3 Results

**Fig. 2:**
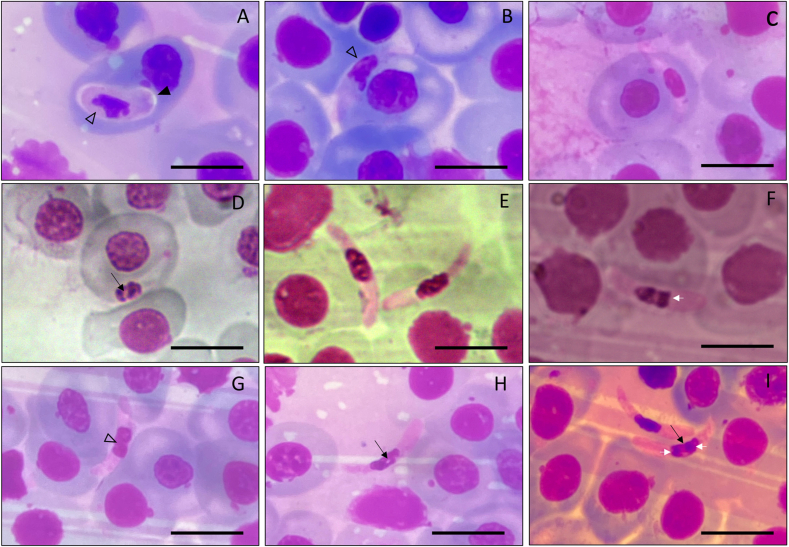
Gamonts intracellular and extracellular of *Hepatozoon* sp. found in the blood smears of *Rhinella marina* from Santarém, Pará state, Brazil. **(A**–**C)** intracellular gamonts; **(C**–**F)** extracellular gamonts. Vacuoles are indicated by a black arrow. Parasitophorous capsule indicated by arrow-head. The nucleus in lobular forms or undefined lobular forms indicated by an empty arrow-head; Chromatin with two concentrations indicated by a white short arrow. Scale bar = 10 μm.

From the eight specimens collected, two were positive for hemoparasites by microscopy, one *R. marina* positive for *Hepatozoon* sp. (Fig. 2) and one *R. diptycha* with a co-infection between *Lankesterella* sp. (Fig. 5) and *Hemolivia* sp. (Fig. 7), with a prevalence of 20% and 33.3% respectively. These parasite genera were identified following available literature (Costa and Pereira, 1971; Lainson and Paperna, 1995; Smith, 1996; Úngari et al., 2021; Chagas et al., 2021b), however, some morphological features combined with molecular data allowed us to describe one new species that are presented below. The mean infection intensity of the extracellular gamonts of *Hepatozoon* was 64 × 10^4^ parasites/mL and intracellular gamonts was 21 × 10^4^ parasites/mL of blood. The mean infection intensity of the sporozoites of *Lankesterella* was 53 × 10^4^ parasites/mL of blood, and for gamonts of *Hemolivia* was 20 × 10^4^ parasites/mL and for meronts of *Hemolivia* was 12 × 10^4^ parasites/mL of blood. The mean measurements obtained from the five specimens of *R. marina* were as follows: SVL of 126.40 ± 9.52 mm (115–141) and W of 176.40 ± 40.01 g (130–240); for the three specimens of *R. diptycha* they were SVL of 127.66 ± 7.50 mm (120–135) and W of 311.66 ± 46.50 g (265–358). No ectoparasite was found in the specimens captured.

**Fig. 3:**
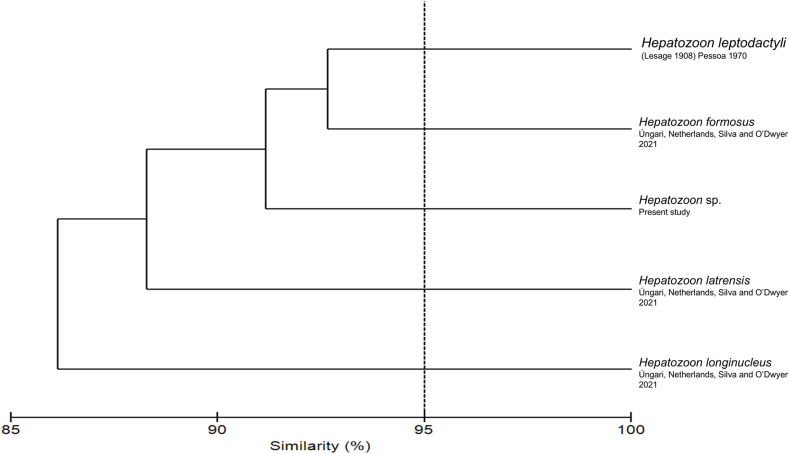
Bray-Curtis similarity analysis, based on the measurements of *Hepatozoon* species valid for Brazil compared to the measurements of *Hepatozoon* sp.

**Fig. 4:**
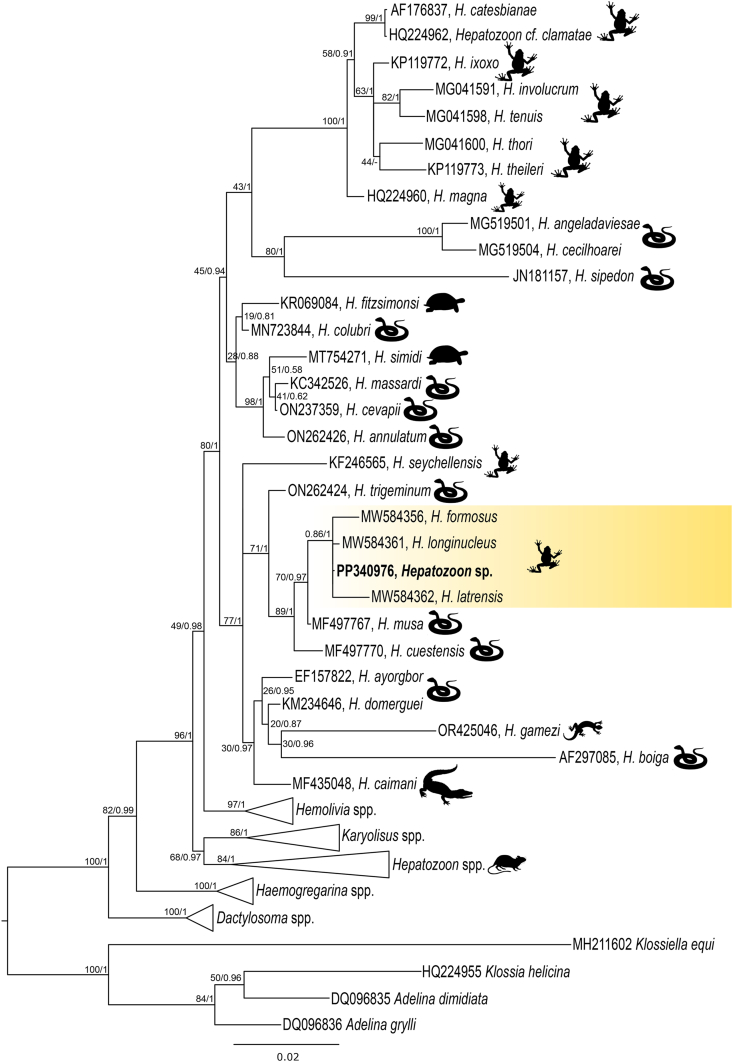
Phylogenetic hypothesis of *Hepatozoon* parasites obtained using maximum likelihood and Bayesian inference from *18S* rDNA sequences 1199 bp. The sequences obtained in this study are provided in bold font. Sequences of *Adelina dimidiata*, *Adelina grylli*, *Klossiella equi* and *Klossia helicina* were used as outgroup. The silhouettes located near the clade nodes indicate the host from which the parasites were isolated: a frog for amphibians, a lizard, a turtle, a caiman, a snake, and a rat for mammals. Bootstrap values and posterior probabilities are shown close to the nodes.

**Fig. 5:**
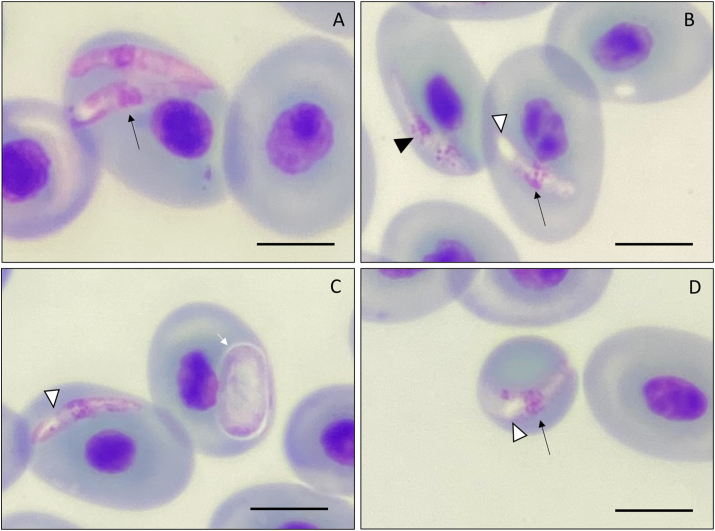
Sporozoites of *Lankesterella oliviacatarinae* n. sp. found in the blood smears of *Rhinella diptycha* from an Industrial area of Marabá, state of Pará, Brazil. **(A**–**D)** sporozoites; **(C)** coinfection between intracellular *Lankesterella oliviacatarinae* n. sp. and *Hemolivia* sp. Granular concentration in one of the magenta-colored poles indicated by a black arrowhead. The presence of vacuoles is indicated by a white arrowhead. Scale bar = 10 μm.

**Fig. 6:**
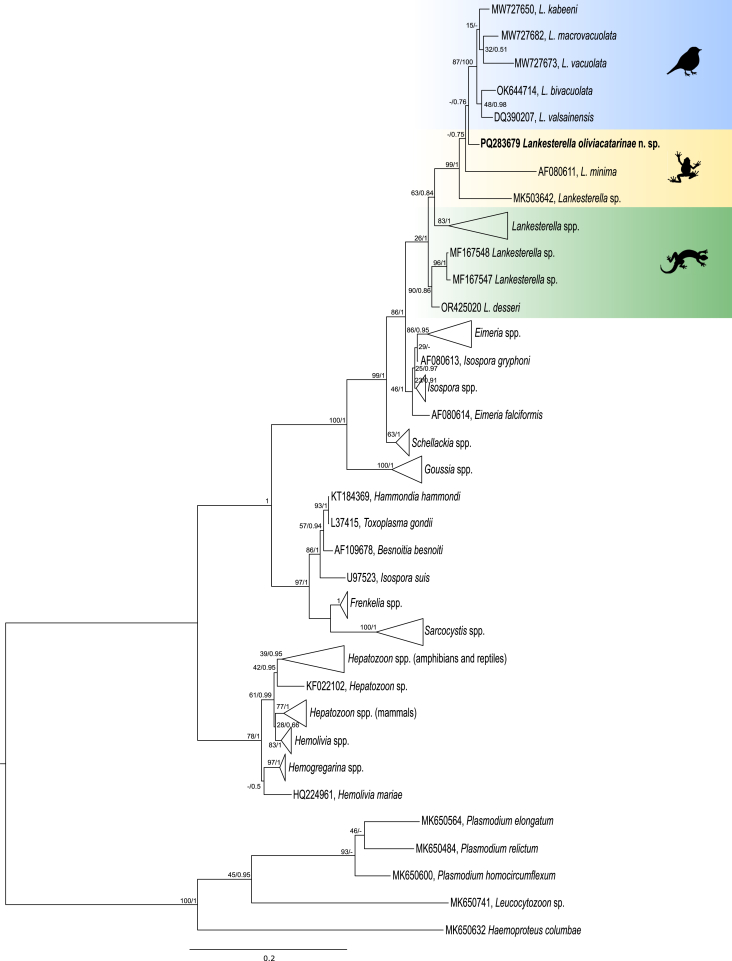
Phylogenetic hypothesis of *Lankesterella* parasites obtained using maximum likelihood and Bayesian inference from *18S* rDNA sequences 1354 bp. The sequences obtained in this study are provided in bold font. Sequences of Haemosporidian (*Plasmodium* spp., *Haemoproteus* sp. and *Leucocytozoon* sp.) parasites were used as outgroup. The silhouettes located near the clades indicate the hosts from which the parasites were isolated: a bird for avian hosts, a frog for amphibians, and a lizard for reptiles. *Lankesterella* sequences from amphibians (in yellow), lizards (in green) and birds (in blue) are highlighted in the tree. Bootstrap values and posterior probabilities are shown close to the nodes.

Phylogenetic analysis of *Hepatozoon* placed the obtained sequence in the same clade as other *Hepatozoon* species of anurans described in Brazil and separated from the species described in lizards and in other zoogeographical regions (Fig. 4). For *Lankesterella,* the obtained sequence is closely related to the species described in birds and amphibians, separated from the species reported in lizards (Fig. 6). None of the obtained sequences had a 100% similarity with other sequences deposited on GenBank. The combination of morphological features and molecular data allowed us to describe a novel species of *Lankesterella*. Additionally, detailed morphological analysis of *Hepatozoon* sp. and *Hemolivia* sp. are presented below.

### 3.1 *Hepatozoon* sp. in *R. marina*

**Intracellular Mature Gamonts (**Fig. 2a–d**)**: showed a robust shape, varying from cylindrical forms with attenuated at the ends (Fig. 2b and c) to reniform capsular forms (Fig. 2a). The parasitophorous capsule was not always evidenced and cytoplasm stained whitish without any cytoplasmic vacuoles; measurements of 15.81 ± 2.58 μm (10.82–19.55; *n* = 20) long and 3.97 ± 0.26 μm (3.65–4.49; *n* = 20) wide. The nucleus has dense chromatin, stained in dark purple, with undefined lobular shapes (Fig. 2a and b), and is located in the central region of the parasite body, with measurements 5.34 ± 1.47 (3.17–8.3; *n* = 20) μm long and 2.89 μm ± 0.18 (2.54–3.17; *n* = 20) wide (Fig. 2a–d.) Intracellular mature gamonts cause displacement of the host cell nucleus and slight morphological changes to the erythrocyte (Fig. 2a) (*U=*4.0, p = 0.041).

**Extracellular Mature Gamonts (**Fig. 2e–i**)**: present a cytoplasmic body, with staining ranging from whitish to light purple, with sausage-shaped forms, the ends show elliptical terminations, with slight curvature in one or both apical regions; measurements of 17.20 ± 0.47 μm (16.44–18.04; *n* = 20) long and 3.06 ± 0.41 μm (2.25–3.59; *n* = 20) wide (Fig. 2e–i). The nucleus shapes varied from cylindrical to elliptical, with the presence of vacuoles (Fig. 2d–h, e), and two to three concentrates (Fig. 2f–i) of chromatin with a strong purple color. The nucleus of the parasite has measurements 5.39 ± 0.88 (3.57–6.61; *n* = 20) μm long and 2.79 μm ± 0.29 (2.25–3.21; *n* = 20) wide. As the parasites were found in the extracellular environment, it was not possible to detect alterations in the structures of the host cells.

Based on morphological and morphometric similarity, as well as analyses using the Bray-Curtis index (Fig. 3), the parasitic forms observed in *Hepatozoon* sp. can be readily distinguish from the other species described in anurans from Brazil: *H. leptodactyli*, *H. formosus*, *H. longinucleus* or *H. latrensis*. Nevertheless, phylogenetic analyses do not provide sufficient evidence to support the description of a new species of *Hepatozoon* in amphibians (Fig. 4 and Table 1).

**Table 1:**
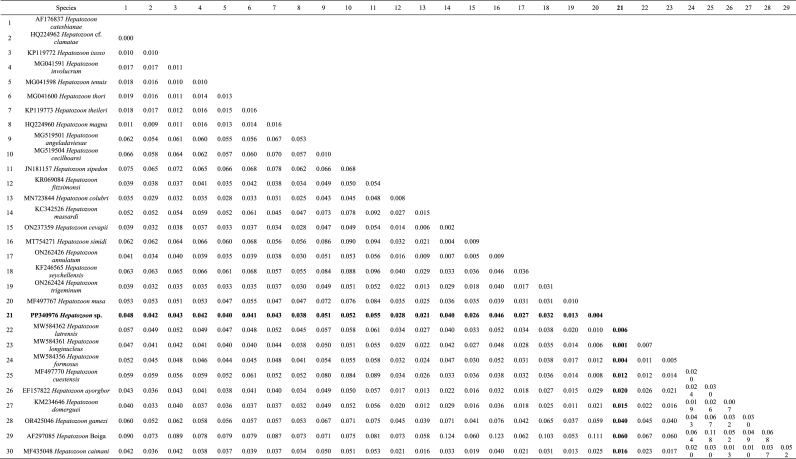
Matrix shows the p-distance (pair-wise distance) of the nucleotide sequences among the *Hepatozoon* sequences from amphibians, reptiles and mammals available at Genbank (1140 nt). Sequence obtained in this study are given in bold.

### 3.2 *Description of Lankesterella oliviacatarinae* n. sp. in *R. diptycha*

#### 3.2.1 *Lankesterella oliviacatarinae* n. sp. Alves-Coêlho, Chagas-de-Souza, Chagas, Gutiérrez-Liberato, André and Viana

**Phylum:** Apicomplexa Levine 1970.

**Class:** Conoidasida Levine 1988.

**Subclass:** Coccidiasina Leuckart 1879.

**Order:** Eucoccidiorida Léger 1911.

**Suborder:** Eimeriorina Léger and Duboscq 1911.

**Family:** Lankesterellidae Nöller 1920.

**Genus:***Lankesterella* Labbé 1899.

**Type-host:***Rhinella diptycha* (Cope, 1862) (Anura: Bufonidae).

**Site of infection:** Erythrocytes in the peripheral blood; no other data.

**Prevalence:** 33% (1 out of 3 examined *R. diptycha* was infected)

**Vector:** Unknown.

**Etymology:** In honor of TAC's daughters, Olívia Brito Coelho and Catarina Brito Coelho, who are his greatest gift, inspiration and purpose for carrying out scientific research in parasitological studies with free-living animals in Brazil.

**Type-locality:** Industrial area of the Municipality Marabá, state of Pará, Brazil (coordinates 5°25′2.83″ S, 49°6′27.87″ W).

**Type-material:** Hapantotype (thin blood smear from *R. diptycha* was deposited in the Coleção Parasitológica, Laboratório de Ecologia e Comportamento Animal (LECAN), Santarém, PA, Brazil [UFOPA-P(Hemo)0010A].

**Mean Infection Intensity:** Sporozoites were 53 × 10^4^ parasites/mL of blood.

**DNA sequence**: *18S* rDNA gene sequence obtained with 1158 bp of length and deposited in GenBank, accession number [PQ283679].

**ZooBank registration**: the Life Science Identifier (LSID) of the article is [zoobank.org:pub:B156E394-F35B-44E4-AF89-A2FF3A21B708] and the LSID for the new name *Lankesterella oliviacatarinae* n. sp. is [zoobank.org:act:FC5E9629-9FA6-4835-8365-95BADD14DA56]

**Note:** The authors of the new taxon are different from the authors of this paper; Article 50.1 and Recommendation 50A of the International Code of Zoological Nomenclature (CZN International Code of Zoological Nomenclature, 1999).

### 3.3 Diagnosis

**Sporozoites (**Fig. 5a–d**):** Slightly sausage body with nucleus varying from centrally located to a polar position (Fig. 5a–d), with one of its extremities having a bigger width than the other (Fig. 5b–d). The sporozoites measured 13.14 μm ± 1.42 (10.72–15.20, *n* = 20) in length, 2.94 μm ± 0.35 (1.81–2.94, *n* = 20) in width and 23.21 μm ± 3.25 (18.77–29.30, *n* = 20) in area of parasite. The parasite nucleus measured 2.71 μm ± 0.42 (1.9–3.59, *n* = 20) in length, 2.24 μm ± 0.36 (1.78–2.24, *n* = 20) in width and 4.55 μm ± 1.31 (2.24–7.47, *n* = 20) in area of parasite nucleus. In some sporozoites, the presence of vacuoles can be observed at one of poles or in a perinuclear position (Fig. 5b–d, white arrowhead). Cytoplasmic body has a coloration varying from hyaline to light magenta, with the presence of granulations (Fig. 5b, black arrowhead). The coinfection with gamonts of *Hemolivia* sp. is observed (Fig. 5c, short white arrow). The nucleus of the infected cell may be slightly dislocated, but in most observations, there is no change in the nuclear positioning of the host cell (*U=* 28.0, p = 0.619).

### 3.4 Remarks

*Lankesterella minima* (Chaussat, 1850), described in *Pelophylax lessonae* (Camerano, 1882) (syn. *Pelophylax* kl. *esculentus*), has sporozoite forms with robust bodies measuring 12–13 μm long and 3–3.5 μm wide (Chaussat, 1850; Tse et al., 1986; Desser et al., 1990). The refractile body was not visible, but there are two vacuoles, one at each pole. The nucleus is medium-sized with the presence of granules. Considering sporozoites of *Lankesterella oliviacatarinae* n. sp., they are similar in size, measuring 10.72–15.20 μm long x 1.81–3.01 μm wide, with an mean of 13.14 μm long x 2.94 μm wide, present a slightly centralized nuclear region (Fig. 5b–d), and may have slightly dispersed chromatin toward one of the body attenuations or perinuclear (Fig. 5d). However, vacuoles were present at the poles and perinuclear (Fig. 5b–d). Morphologically, there is a contrast between the species, considering that *L. oliviacatarinae* n. sp. is a new species of the genus *Lankesterella*.

*Lankesterella petiti*Lainson and Paperna (1995), described in *R*. *marina* (syn. *Bufo marinus*), presents sporozoites measuring 7.0–9.0 μm long and 1.0–1.4 μm wide, with a slender body and often arranged in a banana shape (Lainson and Paperna, 1995). A perinuclear vacuole and a subterminal nucleus are visible. Despite the poor description of the sporozoites, it is clear that compared to *L. oliviacatarinae* n. sp. they are different in shape and size, being crescent-shaped with nuclear chromatin varying from aggregated to dispersed, which is not seen in *L. petiti*, measurements 10.72–15.20 μm long and 1.81–3.01 μm wide, with an mean of 13.14 μm long and 2.94 μm wide. In addition, they have a slightly centralized nuclear region and may have slightly dispersed chromatin toward one of the body attenuations, as well as the presence of a vacuoles.

*Lankesterella alencari*Costa and Pereira (1971), described in *Leptodactylus latrans* (Steffen, 1815) (syn. *Leptodactylus ocellatus*), presents elongated forms measuring 10.79 μm long and 2.4–3.3 μm wide, in addition to the nucleus measuring 0.8 μm in diameter (Costa and Pereira, 1971). It has pale bluish cytoplasm and nuclear material in strands or granules, the granules being weakly stained. A vacuole is visible at only one pole. It is reported to infect young erythrocytes, leukocytes, and large lymphocytes, with multiple infections occurring in these cells. Normally the position of the nucleus is not altered, but hypertrophied erythrocytes are found, including erythrocytes with loss of nucleus. Compared to L. *oliviacatarinae* n. sp. there are differences in size and shape. *Lankesterella oliviacatarinae* n. sp. is larger and the shape of the sporozoites is elongated with a centralized nucleus but with chromatin in granules dispersed perinuclearly or in the cytoplasm (Fig. 5b–d). In L*.oliaviacatarinae* n. sp. the vacuoles are localized in one pole or perinuclear. In L. *oliviacatarinae* n. sp. there is no infection in leukocytes, but there is infection in young erythrocytes, including erythrocytes without nuclei, compatible with *L. alencari*. Finally, the presence of refractile bodies is not reported in *L. alencari*, and some of the forms found vary from ovoid to elongated, and in *L. oliviacatarinae* n. sp. no sporozoites were found in ovoid forms.

*Lankesterella poeppigii*Paperna, Bastien, Chavatte and Landau 2009 was described in *Bufo poeppigii* (Tschudi, 1845) in Peru, reporting merogonic development and oocyst stages in tissues as well as sporozoites in the blood (Paperna et al., 2009). *Lankesterella poeppigii* is recorded with up to three sporozoites infecting the same erythrocyte, measuring 8.7–9.8 μm long and 2.8–3.1 μm wide, with conspicuous nuclear chromatin, but present at the margins of the sporozoites. In addition, the authors consider the refractile body to be slightly visible. The main differences with *L. oliviacatarinae* n. sp. are the slightly body (Fig. 5b and c), and the vacuoles at one pole or perinuclear observed. Parasitism does not result in nuclear displacement of the host cell.

*Lankesterella ptychadeni*Paperna and Ogara (1996) is described in *Ptychadena mascareniensis* (Duméril and Bribon, 1841) found in Aheru rice fields, east of Lake Victoria in Kisumu, Kenya (Paperna and Ogara, 1996). The description of sporozoites in the blood includes slender forms found in monocytes and other leukocytes, measuring 5.6–6.5 μm long and <1.2 μm wide, as well as sporozoites occurring in erythrocytes, which have robust forms measuring 4.2 μm long and 2.8 μm wide or slender forms measuring 5.6–7.7 μm long and 1.4 μm wide. They are described as occupying a larger portion of the host cell cytoplasm, with a pale blue staining refractile body. *Lankesterella ptychadeni* have sporozoites, with morphological differences when compared with *L. oliviacatarinae* n. sp., with a slightly body and the presence of vacuoles. In addition, the size of body and nucleus of *L. oliviacatarinae* n. sp. is considerably larger when compared with *L. ptychadeni*.

*Lankesterella dicroglossi*Paperna and Ogara (1996), described in *Dicroglossus occipitalis* (Gunther, 1858) found in Lake Baringo, Kenya, consisting of sporozoites, but no detailed description on erythrocytes, only some information such as having an anterior and posterior refractile body and measuring 7–9 μm long and 1–1.4 μm wide (Paperna and Ogara, 1996). According to the description, *L. oliviacatarinae* n. sp. differs in size, shape and presence of vacuoles from *L. dicroglossi*. In addition, the chromatin of the nucleus granulated in the cytoplasm of the parasite is evident in *L. oliviacatarinae* n. sp. (Fig. 5b).

*Lankesterella bufonis*Mansour and Mohammed (1962) is described in *Sclerophrys regularis* (Reuss, 1833) (syn. *Bufo regularis*) collected in the Dahshour area of Giza Province, Egypt (Mansour and Mohammed, 1962). The forms found in the blood are described as small, slender sporozoites measuring 6.2–12.8 μm long and 0.7–2.0 μm wide, with an mean of 10.3 long μm and 1.2 μm wide. A slight curvature gives these bodies an open crescent shape with a regular, smooth outline and one anterior end more pointed than the other. The nucleus is either terminal or, more commonly, subterminal and always posterior to the third part of the body and is characterized as a small, oval and delicate structure measuring 1.1–2.6 μm long and 0.9–1.7 μm wide, with an average of 2 μm long and 1.4 μm wide. The vacuoles are single, rounded and located in the central part, immediately in front of the nucleus, with a few dark colored granules sometimes seen around the vacuole. Compared to L. *oliviacatarinae* n. sp., sporozoites are considered different in shape and size, presenting crescent-shaped forms with nuclear chromatin varying from aggregated to granulated dispersed in cytoplasm, which is not seen in *L. bufonis*. The sporozoite nucleus of *L. oliviacatarinae* sp. n. is larger and also has a slightly centralized nuclear region and may have slightly dispersed chromatin toward one of the body attenuations. In addition, vacuoles are observed at one pole or perinuclear, which is not seen in *L. bufonis*.

*Lankesterella canadensis*Fantham, Porter and Richardson 1942, described in *Aquarana catesbeiana* (Shaw, 1802) (syn. *Rana catesbeiana*) caught in Montreal, Quebec and Lake Manitou, Ontario, both in Canada (Fantham et al., 1942). The forms found at both sites were similar, being crescent to bean-shaped with deeply stained cytoplasm. Vacuoles are present at each pole and the nucleus is distinct, sometimes granular, with chromatin granules found in the nuclear membrane. They are large parasites, ranging from 10.6 to 19.2 μm long and 3–7.8 μm wide and extracellular forms are found. As described, *L. oliviacatarinae* n. sp. differs in size, shape and the presence of a vacuoles in perinuclear. In addition, the presence of two vacuoles, one at each pole, is a striking difference between *L. canadensis* and *L. oliviacatarinae* n. sp. Extracellular forms were not found in *L. oliviacatarinae* n. sp. Finally, the size of *L. canadensis* is larger in both length and width than that found in *L oliviacatarinae* n. sp.

*Lankesterella hylae*Cleland and Johnston, 1910, described in *Litoria caerulea* (White 1710) (syn. *Hyla caerulea*) from Sydney, Australia, presents 9–11 μm long x 3 μm wide (Cleland and Johnston, 1910). The intraerythrocytic form found with an open crescent shape and a concavity facing the host nucleus. The main characteristic of *L. hylae* is the presence of an extensive bulge immediately posterior to the nucleus, which may or may not be stained and is only seen in heavily infected hosts. The color of the parasite was pale blue and there were usually fine reddish granules. The nucleus was closer to one end than the other, with a banded appearance, and part of the parasite apart from the nucleus was non-granular, but the rest of the parasite was clearly granular. There is a vacuole associated with the nucleus. Displaced host cell nuclei were observed, but in no case where they enlarged or distorted, as is often seen in infections with other reptile and amphibian hemogregarines (e.g., *Hepatozoon* spp.). In addition, double and triple infections were common, and in double infections the parasites were side by side, parallel to each other, forming an open V-shape. In view of the information provided, the shape of the sporozoite found differs from *L. oliviacatarinae* n. sp. because, considering the high infection rate found, no parasite with a bulge was seen. In addition, *L. hylae* is relatively short in comparation with *L. oliviacatarinae* n. sp. The dispersed chromatin in one of the attenuations observed in *L. hylae* corroborates our findings, but the nucleus of *L. oliviacatarinae* n. sp. is centralized, unlike *L. hylae* in which it is subterminal. Finally, there is no refractile body in *L. hylae* and in *L. oliviacatarinae* n. sp. However, in *L. oliviacatarinae* n. sp. vacuoles at one pole or perinuclear are observed.

### 3.5 Phylogenetic remarks

Phylogenetic analysis of a fragment of the *18S* rDNA, showed that *L. oliviacatarinae* n. sp. is closely related to avian *Lankesterella* species, and to *L. minima*, a known species of this parasite in amphibians (Fig. 6). The *18S* rDNAsequence of *L. oliviacatarinae* n. sp. is ∼6% different from *L. minima* and ∼7% from *Lankesterella* sp. (GenBank accession number MK503642) (Table 2). The lack of genetic sequences of other *Lankesterella* species infecting anurans did not allow us to genetically in a complete way, but we did observe the formation of clades from different vertebrate groups (anurans, reptiles and birds). However, the detailed morphological analysis conducted in this study, allowed to describe a new species of *Lankesterella* in the Brazilian Amazon.

**Table 2:** Matrix shows the p-distance (pair-wise distance) of the nucleotide sequences among the *Lankesterella* sequences from birds, amphibians and reptiles available at Genbank (1354 nt). Sequence obtained in this study are given in bold.

	Species	1	2	3	4	5	6	7	8	9	10	11	12	13	14	15	16	17	18	19	20	21	22	23	24
1	MW727650 *Lankesterella kabeeni*																								
2	MW727682 *Lankesterella macrovacuolata*	0.02																							
3	MW727673 *Lankesterella vacuolata*	0.03	0.04																						
4	OK644714 *Lankesterella bivacuolata*	0.03	0.03	0.04																					
5	DQ390207 *Lankesterella valsainensis*	0.02	0.03	0.03	0.02																				
6	**PQ283679 *Lankesterella oliviacatarinae* n. sp.**	**0.03**	**0.03**	**0.04**	**0.03**	**0.03**																			
7	AF080611 *Lankesterella minima*	0.07	0.07	0.08	0.07	0.07	**0.06**																		
8	MK503642 *Lankesterella* sp.	0.08	0.08	0.10	0.08	0.08	**0.07**	0.10																	
9	MF167551 *Lankesterella* sp.	0.06	0.06	0.07	0.06	0.06	**0.05**	0.09	0.10																
10	MF167550 *Lankesterella* sp.	0.06	0.06	0.07	0.06	0.07	**0.05**	0.09	0.11	0.03															
11	MF167553 *Lankesterella* sp.	0.07	0.07	0.08	0.08	0.08	**0.07**	0.10	0.12	0.04	0.04														
12	KU180248 *Lankesterella* sp.	0.06	0.06	0.07	0.06	0.06	**0.05**	0.08	0.10	0.04	0.04	0.05													
13	OR425019 *Lankesterella desseri*	0.06	0.06	0.07	0.06	0.06	**0.05**	0.09	0.10	0.04	0.04	0.05	0.02												
14	MF167544 *Lankesterella* sp.	0.06	0.06	0.07	0.07	0.06	**0.05**	0.09	0.10	0.04	0.04	0.05	0.02	0.03											
15	MF167552 *Lankesterella* sp.	0.06	0.06	0.07	0.06	0.06	**0.05**	0.09	0.10	0.04	0.04	0.05	0.02	0.03	0.00										
16	MF167555 *Lankesterella* sp.	0.06	0.06	0.07	0.06	0.06	**0.05**	0.09	0.10	0.04	0.04	0.05	0.02	0.03	0.00	0.00									
17	MF167554 *Lankesterella* sp.	0.05	0.06	0.07	0.07	0.06	**0.05**	0.08	0.10	0.04	0.04	0.05	0.02	0.02	0.01	0.01	0.01								
18	KJ131417 *Lankesterella* sp.	0.07	0.07	0.08	0.07	0.07	**0.06**	0.10	0.10	0.05	0.05	0.06	0.04	0.04	0.03	0.03	0.03	0.02							
19	MF167545 *Lankesterella* sp.	0.06	0.06	0.07	0.07	0.07	**0.05**	0.09	0.10	0.04	0.04	0.06	0.03	0.04	0.03	0.03	0.03	0.03	0.04						
20	MF167546 *Lankesterella* sp.	0.06	0.06	0.07	0.07	0.07	**0.05**	0.09	0.10	0.04	0.04	0.05	0.03	0.03	0.03	0.03	0.03	0.03	0.04	0.01					
21	MF167549 *Lankesterella* sp.	0.06	0.06	0.07	0.06	0.06	**0.05**	0.08	0.10	0.04	0.04	0.05	0.03	0.04	0.03	0.03	0.03	0.03	0.04	0.02	0.02				
22	MF167547 *Lankesterella* sp.	0.05	0.05	0.06	0.06	0.06	**0.05**	0.08	0.09	0.05	0.05	0.06	0.04	0.04	0.04	0.04	0.04	0.04	0.05	0.04	0.04	0.05			
23	MF167548 *Lankesterella* sp.	0.05	0.05	0.06	0.06	0.06	**0.05**	0.08	0.08	0.05	0.05	0.06	0.04	0.04	0.04	0.04	0.04	0.04	0.05	0.04	0.04	0.04	0.00		
24	OR425020 *Lankesterella desseri*	0.05	0.05	0.06	0.06	0.06	**0.04**	0.08	0.08	0.04	0.04	0.06	0.04	0.04	0.04	0.04	0.04	0.04	0.05	0.04	0.04	0.04	0.02	0.02	

### 3.6 Forms of *Hemolivia* sp. in *R.diptycha*

**Fig. 7:**
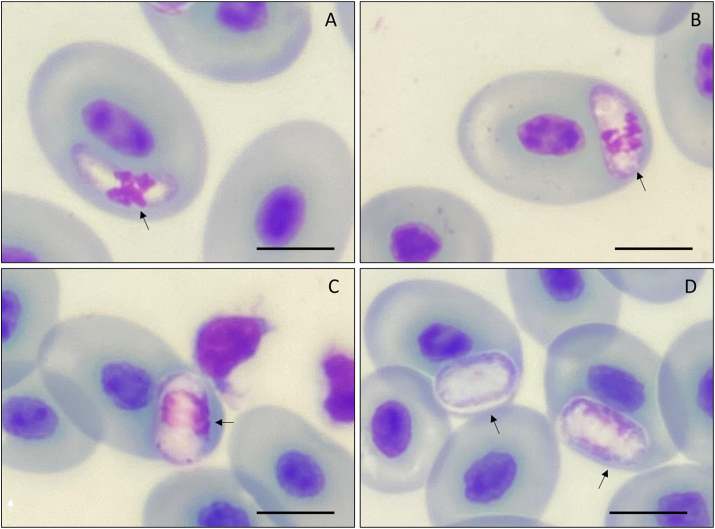
Blood stages of *Hemolivia* sp. found in the blood smears of *Rhinella diptycha* from Marabá, Pará state, Brazil. **(A)** intracellular meronts; **(B**–**C)** young gamonts. **(D)** encapsulated mature gamonts. Scale bar = 10 μm.

**Meronts** (Fig. 7a): Thin capsular shape measuring 9.59 ± 0.27 μm (9.18–9.91, *n* = 20) long and 3.53 ± 0.21 μm (3.25–3.80, *n* = 20) wide, with predominantly hyaline cytoplasm and rare vacuoles. Nucleus with marked polar displacement measuring 2.43 ± 0.04 μm (2.38–2.49, *n* = 20) long and 3.19 ± 0.05 μm (3.14–3.30, *n* = 20) wide, dense chromatin with an amorphous appearance.

**Young Gamonts** (Fig. 7b and c): Capsular shape varied from robust to slightly reniform, measuring 14.53 ± 0.96 μm (13.20–15.98, *n* = 20) long and 4.61 ± 0.16 μm (4.36–4.80, *n* = 20) wide, with hyaline cytoplasm and the presence of vacuoles. Nucleus with dense chromatin in lateralization movement measuring 4.27 ± 0.18 μm (3.97–4.53, *n* = 20) long and 4.97 ± 0.22 μm (4.69–5.29, *n* = 20) wide, suggesting that it is a folded on itself. The nucleus is fragmented.

**Encapsulated Mature Gamonts (**Fig. 7d**):** General capsular shape with hyaline centroid measuring 9.68 ± 0.60 μm (8.95–10.23, *n* = 20) long and 6.26 ± 0.43 μm (5.62–6.58, *n* = 20) wide. It is lightly marked with dispersed chromatin at the longitudinal ends. It is completely encapsulated, with no marked presence of a nucleus or vacuoles.

## 4 Discussion

The key result of this study is the description of one parasite species infecting anurans in the Brazilian Amazon: *L. oliviacatarinae* n. sp., infecting *R. diptycha* which can be readily distinguished by morphology and morphometry from the other *Lankesterella* species described in anurans of Brazil and the world. These findings are supported by the phylogenetic analysis and the genetic distance of the*18S* rDNA fragments of *L. minima* and *Lankesterella* sp.(both found in anurans) when compared to *L. oliviacatarinae* n. sp.

To the best of our knowledge, this is the first study to combine molecular, morphological and morphometric data to identified *Hepatozoon* in *R. marina* and *Lankesterella* in *R. diptycha*. This report emphasizes the limited understanding of the biodiversity of hemoparasites in amphibians in Brazil and provides valuable data that can be used in future studies to describe this biodiversity.

*Hepatozoon* spp. infections have been reported in a wide variety of vertebrates (Metzger et al., 2008; Pinto et al., 2013; Merino et al., 2014; Soares et al., 2017; Úngari et al., 2018; de Castro Demoner et al., 2019; Perles et al., 2019; De Paula et al., 2022; Picelli et al., 2023) and are found in anuran hosts in Brazil (Cunha and Muniz, 1927; Costa et al., 1973; Kattar, 1986; Leal et al., 2009; Menezes Leal et al., 2015; Ferreira et al., 2020; Pinho et al., 2021; Úngari et al., 2021; Bilhalva et al., 2023). However, data on *Hepatozoon* spp. species infecting anurans from Amazonian are still scarce in the literature, with most of the records presenting only morphological features of the parasites, but without morphometric analysis.

Overall, a few studies have recorded *Hepatozoon* species using an integrative taxonomy in the world, which combines morphological, morphometric, and molecular data (Barta et al., 2012). Until recently, only *H. leptodactyli* had been formally described in Brazil based on morphological and morphometric aspects (Costa et al., 1973). Furthermore, previous studies recording the occurrence of *Hepatozoon* in the blood of amphibians have used a substantial number of amphibians that were killed for the purpose of obtaining their samples (Sousa and Borriello Filho, 1974; Kattar, 1986; Menezes Leal et al., 2015). Similarly to other studies (Netherlands et al., 2017; Úngari et al., 2020), the present study also shows that it is possible to sample amphibians and release them in nature after the procedure. However, for some studies, euthanasia is the only solution to complete the analysis and obtain reliable results. For example, studies on the taxonomy of helminths, descriptions of life cycles, merogony data of apicomplexes and others. The discussion is essential, mainly because in the past 30 years, the concerned regarding the decline of amphibian population increased, with studies identifying habitat loss, climate change, and emerging diseases as major drivers (Wake and Vredenburg, 2008; Marcogliese et al., 2009; Vredenburg et al., 2010). Being able to investigate their pathogens without compromising their survival in nature is essential for future studies.

The most recent study in Brazil describing new species of *Hepatozoon* spp. in anurans was performed by Úngari et al. (2021), who used morphological, morphometric, histological and molecular data to describe the following species: *H. latrensis*, *H. longinucleus* and *H. formosus*, which were found in anuran species of the family Leptodactylidae in the state of Mato Grosso, Brazil. Nevertheless, the recorded species show morphological and morphometric data variations compared to *Hepatozoon* sp. Regarding molecular data, the *Hepatozoon* sp. detected in the present study and classified within the Brazilian clade of amphibian *Hepatozoon* spp. exhibits interspecific divergences from the other species. Nevertheless, it was not possible to confirm that we are dealing with a new *Hepatozoon* species at this time, given the limitations of the available tools, and the morphological and morphometric plasticity of parasites belong to the *Hepatozoon* genus, which is already known (Pessoa and Cavalheiro, 1969; O'Dwyer et al., 2013; De Paula et al., 2022). We consider it more parsimonious, even with the possibility of describing it as a new species, to treat the finding of this study as *Hepatozoon* sp. We recommend that further studies in the sampling area and with the same host species would be conducted in the near future, as well as the application of molecular investigation targeting other genes.

In another recent study, Ferreira et al. (2020) provided only molecular data, excluding any morphological or morphometric analysis, found a higher prevalence of hemogregarines in anuran species of Leptodactylidae compared to Bufonidae. However, in the study by Úngari et al. (2021), when describing the species *H. latrensis* found in *L. latrans*, was observed to have 100% genetic similarity with sequences in the study by Ferreira et al. (2020), indicating that the *18S* rDNAsequences corresponded indeed to *H. latrensis*.

Compared to the *Hepatozoon* species described in anurans and snakes in Brazil, our study reveals remarkable morphological differences, along with phylogenetic aspects that distinguish the Brazilian anuran *Hepatozoon* group from the species described in Brazilian snakes (Fig. 4). The close relationship between anurans and snakes is well known and is mainly related to the fact that anurans are an important paratenic host in the *Hepatozoon* spp. cycle of some reptiles (Lainson et al., 2003; Viana et al., 2012; Ferreira et al., 2020).

Regarding the descriptions of hemogregarine species of the *Lankesterella* genus for Brazilian anurans (Costa and Pereira, 1971; Lainson and Paperna, 1995), they are established through the observation of morphological, histological, and ultrastructural characteristics. These factors are important for the characterization of the different forms of development of the parasite in its anuran host. However, the combination of different methodologies for species description is essential. This approach is necessary due to the gaps found in the descriptions of valid species of anuran parasites in the genus *Lankesterella*, which highlight the importance of incorporating phylogenetic analyses to support both morphological and morphometric data. In this context, it was unnecessary to euthanize the individuals studied for histological analysis to differentiate between *Lankesterella* and *Schellackia* (Desser, 1993; Lainson and Paperna, 1995). These two were distinguished based on morphological aspects seen in our study and supported by phylogenetics.

This is the second time that *Lankesterella* has been detected in *R. diptycha* by molecular method in Brazil (Ferreira et al., 2020). In our study, we presented morphological, morphometric, and molecular data to support the identification of a new species for this genus. Notably, *L*. *oliviacatarinae* n. sp. is closely related to the *Lankesterella* spp. species reported in birds (Chagas et al., 2021a, 2021b) (Fig. 6). This suggests a vectorial action that should be the target of more robust studies that include experimental infection cycles with different hematophagous invertebrates. Nevertheless, it seems probable that *L. oliviacatarinae* n. sp. is not part of a clade comprising amphibians, nor can it be placed alongside *L. minima*, given the lack of available genetic sequences. This highlights the need for further investigation into the diversity of parasites within this genus affecting amphibians not only in Brazil, but in a worldwide scale. Furthermore, although *Lankesterella* and *Schellackia* are closely related genera (Megía-Palma et al., 2017, 2018), the recovery of phylogenetic relationships has become clearer with increasing availability of molecular data. It should be noted that both morphologically and phylogenetically, the two genera are effectively distinguished.

Our study provides information on mean infection intensity, although to obtain an accurate estimate of the mean infection intensity in hosts at a given location, a larger sample of individuals needs to be examined. Unfortunately, this information is not presented in most of the studies describing the different *Lankesterella* species, making any comparisons in this sense impossible (Cleland and Johnston, 1910; Fantham et al., 1942; Mansour and Mohammed, 1962; Lainson and Paperna, 1995; Paperna and Ogara, 1996). The present study revealed a high infection intensity of *Lankesterella* and, to a lesser extent, of *Hemolivia* sp. Despite efforts to obtain molecular data on *Hemolivia* sp. in the *R. diptycha* sample, only *Lankesterella* sequences were amplified. This might be due to the higher *Lankesterella* infection intensity in comparison to *Hemolivia*, a higher affinity between the primers with *Lankesterella* DNA. This is not new in studies with hemoparasites of animals. For example, it is known that for avian haemosporidian parasites, some primers (including the most frequently used ones) fail to amplify the parasites' DNA for certain host groups, as well as certain combinations of mixed infections. (Bernotienė et al., 2016). However, such detailed studies have never been done with blood parasites of amphibians and available primers, which should be encouraged in the future.

Furthermore, the application of traditional primers (Hep300/Hep900; Hemo1/Hemo2) resulted in the detection of only *Lankesterella*, suggesting that cloning, targeting other genes, and designing new primers may be potential solutions to address this intrinsic problem and limitation of the available protocols. In the light of these findings, it is suggested that using only molecular methods to diagnose blood parasite infections in amphibians can result in false-negative results. Therefore, the combination of different techniques is essential to provide a broader view of the infections, as well as more reliable results in terms of parasite identification and their diversity. However, classical parasitology, such as morphological and morphometrical identification of parasites, has been left aside with most of the recent studies focusing only on molecular techniques. It is necessary to train the future generation of parasitologists, so this knowledge will not be lost (Bradbury et al., 2022).

While *Lankesterella* sporozoites are morphologically similar to *Hepatozoon* spp. gamonts, but different in chromatin dispersed in cytoplasm, no traditional nucleus displacement of host cell combined with the molecular data confirms that we were dealing with *Lankesterella* parasites. Morphological and morphometrical analysis also confirmed that this is a new species of *Lankesterella*. But we did not address any aspects of the life cycle of *Lankesterella* in amphibians, which continues to be an unsolved issue. For that, it would be necessary not only to conduct experimental infections with potential vectors, but also to collect infected individuals and to conduct histological examinations. This should be considered in future studies not only in Brazil and with amphibians, but also in other vertebrate groups and localities.

In the context of describing *Lankesterella* species from amphibians, are not always report in details infection in erythrocytes (Lainson and Paperna, 1995). However, there are descriptions of sporozoites found in leukocytes of anurans (Paperna and Ogara, 1996), as reported in birds (Chagas et al., 2021a, 2021b), which were not found in our study, even with the high infection intensity found. This highlights the need for integrative taxonomy to describe new species, emphasizing detailed morphological, morphometric and molecular aspects.

In conclusion, the diversity of hemoparasites of anuran hosts in the Brazilian Amazon is still underestimated, especially if we consider the almost 1200 described species of anurans occurring in Brazil (Segalla et al., 2021). In our study, we present the detection of *Hepatozoon*, *Lankesterella* and *Hemolivia* in bufonids using an integrative approach, describing a new parasite species in amphibians, *Lankesterella oliviacatarinae* n. sp., the second species of the genus *Lankesterella* described with molecular aspects in the world, highlighting and encouraging new studies and descriptions. In addition, in this study, we document a novel occurrence of *Hemolivia* sp. in the Brazilian Amazon, identifying *R. diptycha* as a new host species and the municipality of Marabá, situated in the southeastern region of the state of Pará, as a new site of occurrence. We could also confirm the urge of developing and using different molecular protocols for the identification of blood parasites in anurans.

## CRediT authorship contribution statement

**Tássio Alves-Coêlho:** Writing – review & editing, Writing – original draft, Resources, Methodology, Formal analysis, Conceptualization. **Darlison Chagas-de-Souza:** Writing – review & editing, Writing – original draft, Validation, Methodology, Conceptualization. **Carolina Romeiro Fernandes Chagas:** Writing – review & editing, Visualization, Supervision, Methodology, Formal analysis. **Germán Alfredo Gutiérrez-Liberato:** Writing – review & editing, Validation, Supervision, Methodology. **Lívia Perles:** Writing – review & editing, Methodology. **Amir Alabi:** Writing – review & editing, Methodology. **Marcos Rogério André:** Writing – review & editing, Validation, Supervision, Methodology. **Lúcio André Viana:** Writing – review & editing, Supervision, Resources, Conceptualization.

## Data availability

All data are included in the article.

## Ethics approval

This study was approved by the Animal Use Committee of the Universidade Federal do Amapá (authorization # 011/2021). The licenses for the collection of amphibians used in the present study were granted by the Brazilian Institute for the Environment and Renewable Natural Resources through authorizations issued by the Biodiversity Information and Authorization System - SISBIO nº 77466-1. For access to genetic heritage, this study was authorized by the National System for the Management of Genetic Heritage and Associated Traditional Knowledge (protocol # AC5C1B1).

## Funding

Not applicable.

## Declaration of competing interest

The authors declare that they have no known competing financial interests or personal relationships that could have appeared to influence the work reported in this paper.
